# Tumor Cell-Derived Microvesicles Induced Not Epithelial-Mesenchymal Transition but Apoptosis in Human Proximal Tubular (HK-2) Cells: Implications for Renal Impairment in Multiple Myeloma

**DOI:** 10.3390/ijms18030513

**Published:** 2017-02-27

**Authors:** Aiqi Zhao, Fancong Kong, Chun-Jie Liu, Guoxin Yan, Fei Gao, Hao Guo, An-Yuan Guo, Zhichao Chen, Qiubai Li

**Affiliations:** 1 Institute of Hematology, Union Hospital, Tongji Medical College, Huazhong University of Science and Technology, Wuhan 430022, China; zhao_aiqi@163.com (A.Z.); konglinda212@163.com (F.K.); dysrmygx@sina.com (G.Y.); gaof@hust.edu.cn (F.G.); gh273397674@163.com (H.G.); 2 Department of Hematology, The First Affiliated Hospital of Nanchang University, Nanchang 330000, China; 3 Hubei Bioinformatics & Molecular Imaging Key Laboratory, Department of Bioinformatics and Systems Biology, Key Laboratory of Molecular Biophysics of the Ministry of Education, College of Life Science and Technology, Huazhong University of Science and Technology, Wuhan 430074, China; samliu@hust.edu.cn (C.-J.L.); guoay@hust.edu.cn (A.-Y.G.)

**Keywords:** microvesicles, multiple myeloma, renal impairment

## Abstract

Renal impairment (RI) is one of the hallmarks of multiple myeloma (MM) and carries a poor prognosis. Microvesicles (MVs) are membrane vesicles and play an important role in disease progression. Here, we investigated the role of MVs derived from MM cells (MM-MVs) in RI of MM. We found that MM-MVs significantly inhibited viability and induced apoptosis, but not epithelial-mesenchymal transition in human kidney-2 (HK-2), a human renal tubular epithelial cell line. The protein levels of cleaved caspase-3, 8, and 9, and E-cadherin, were increased, but vementin levels were decreased in the HK-2 cells treated with MM-MVs. Through a comparative sequencing and analysis of RNA content between the MVs from RPMI8226 MM cells (RPMI8226-MVs) and K562 leukemia cells, RPMI8226-MVs were enriched with more renal-pathogenic miRNAs, in which the selective miRNAs may participate in the up-regulation of the levels of cleaved caspase-3. Furthermore, the levels of CD138+ circulating MVs (cirMVs) in the peripheral blood were positively correlated with the severity of RI in newly-diagnosed MM. Our study supports MM-MVs representing a previously undescribed factor and playing a potential role in the development of RI of MM patients, and sheds light on the potential application of CD138+ cirMV counts in precise diagnosis of RI in MM and exploring MM-MVs as a therapeutic target.

## 1. Introduction

Kidney damage from multiple myeloma (MM) is a cause of renal impairment (RI) in patients with MM and a hallmark of MM. As one of the most common complications of MM, RI presents in 20%~25% of newly-diagnosed patients [1,2,3] and more than 50% of advanced disease [4], and carries a poor prognosis [4,5,6].

In RI of MM patients, cast nephropathy is the most common kind of kidney damage. The characteristic renal lesion is a chronic tubulointerstitial nephropathy with marked tubular atrophy and laminated intratubular casts, which was caused by the proximal tubular endocytosis of immunoglobulin light chains [7]. However, in spite of the use of novel drugs to treat MM and novel treatment to treat RI, the results of these treatments to prevent or reverse myeloma nephropathy have been disappointing in some patients [8]. Studies to reveal the novel mechanisms underlying RI of MM patients are urgently needed.

A growing body of studies have confirmed microvesicles (MVs) to play an important role in the interactions between different cells [9]. These tiny particles present a wide range of sizes (100–1000 nm in diameter), are composed of a selection of proteins, lipids, and nucleic acids [10], and have been involved into numerous pathogenic processing of cancer development, such as proliferation, metastasis, angiogenesis, and immunity [11,12,13]. Our previous studies have confirmed the presence of MM-MVs and demonstrated their role in MM-related angiogenesis and tumor cell proliferation [11,14], but it remains unknown about their role in RI of MM. Thus, in the present study, we used MM-MVs to treat human proximal tubular (human kidney-2, HK-2) cells to observe their changes in proliferation, apoptosis, and epithelial-mesenchymal transition (EMT) in vitro, and clinically investigated the associations between peripheral circulating CD138+ cirMV counts and severity of RI in patients with MM.

## 2. Results

### 2.1. Validation and Characteristics of Myeloma Cell-Derived Microvesicles ( MM-MVs)

**Figure 1:**
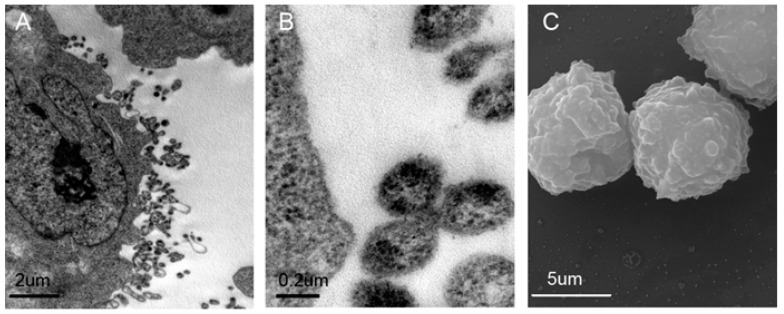

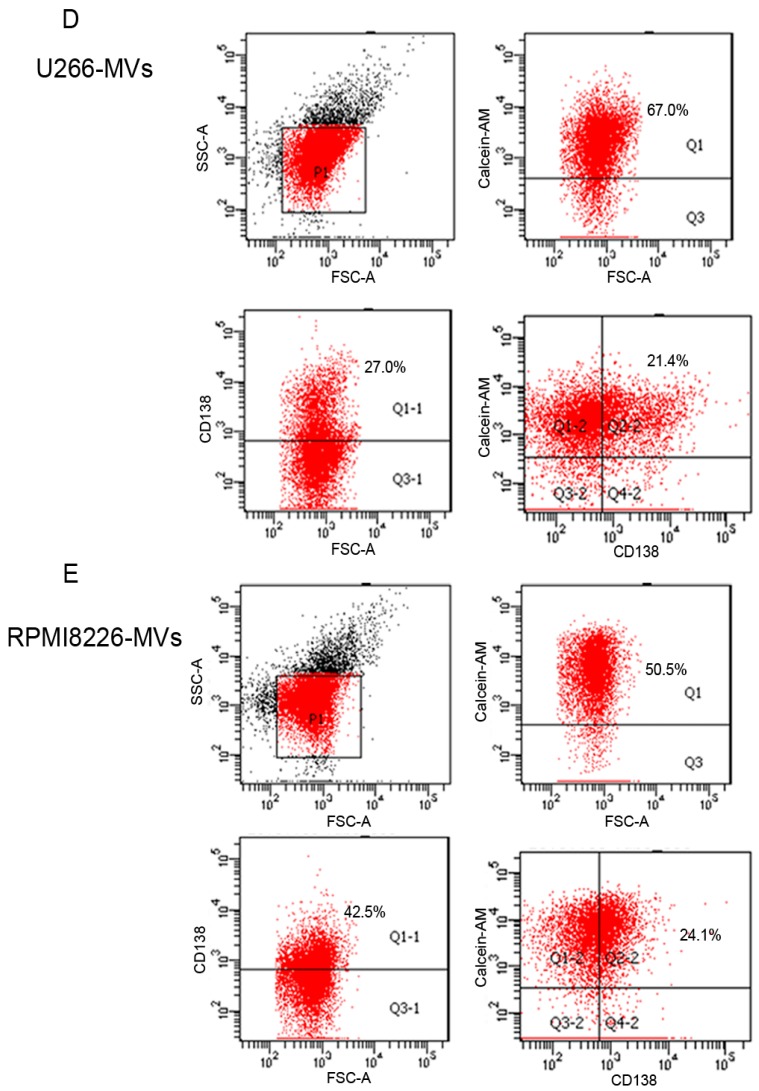
Characterization of myeloma cell-derived microvesicles (MM-MVs) (**A**,**B**) Transmission electron microscopy revealing MVs (arrow) as 100–1000 μm vesicles shed from U266 cells (U266-MVs). Scale bar = 2 μm (**A**) and 0.2 μm (**B**); (**C**) Scanning electron microscopy showing typical morphology of MVs derived from RPMI8226 myeloma cells (RPMI8226-MVs). Scale bar = 2 μm; (**D**,**E**) Representative flow cytometry analysis of U266-MVs and RPMI8226-MVs revealing the presence of CD138+Calcein-AM+ vesicles, with the 1 μm microbeads for gating the MVs for size verification and Calcein-AM to detect the integrity of MVs.

MVs derived from MM cells were acquired and validated as previously described [11,14]. As shown in Figure 1A,B, under a transmission electron microscopy (TEM), U266 MM cells were surrounded by lipid-bilayer vesicles shaping the outlines of smooth curved surfaces, and in various sizes, between 100–1000 nm in diameter. Meanwhile, observed by scanning electron microscopy (SEM), multitudinous diverse spheroid shapes, with diameters in the range of 100–1000 nm, were decorated on the RPMI8226 cell surface (Figure 1C). Furthermore, the MM-MVs derived from U266 cells (U266-MVs) and from RPMI8226 cells (RPMI8226-MVs) were analyzed using flow cytometry. As we previously described, these MM-MVs were determined to express Calcein-AM and CD138 (Figure 1D,E).

### 2.2. MM-MVs Inhibit Viability and Induce Apoptosis in Human Kidney-2 Cells (HK-2 Cells)

**Figure 2:**
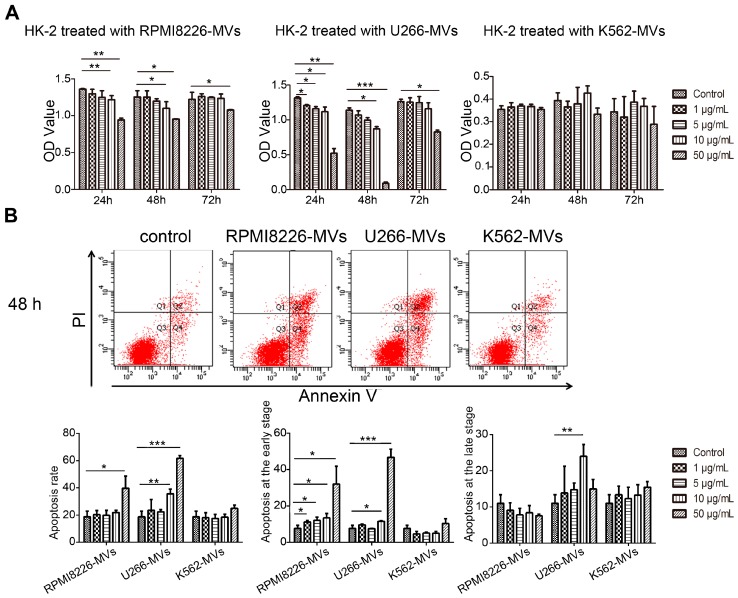

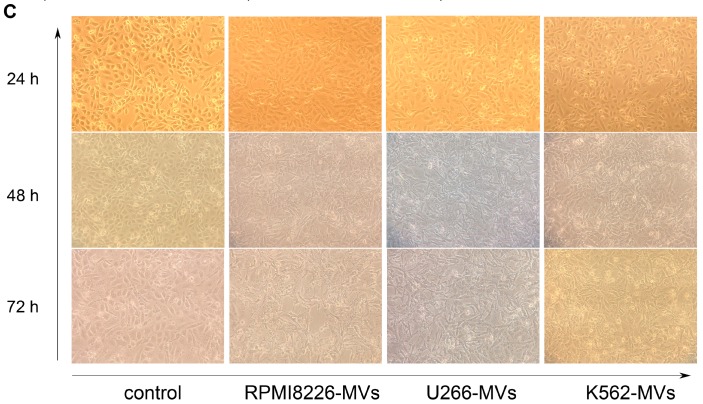
Inhibited viability and induced apoptosis in human kidney-2 cells (HK-2 cells) by MM-MVs. (**A**) HK-2 cells (10^5^/mL) were treated with various concentrations of MM-MVs and K562-MVs (1, 5, 10, and 50 μg/mL) for 24, 48, and 72 h, respectively. The effects of MM-MVs on viability in HK-2 cells were determined with Cell Counting Kit-8 (CCK-8) assay. MM-MVs, but not K562-MVs, inhibited proliferation in HK-2 cells at the time points in a dose-dependent manner; (**B**) After 48 h, the effects of MM-MVs and K562-MVs on apoptosis in HK-2 cells were determined with FITC-annexin V-PI FCM analysis. MM-MVs, but not K562-MVs, induced apoptosis in HK-2 cells at 48 h in a dose-dependent manner. Representative FCM analysis of early and late apoptosis in HK-2 cells treated with MM-MVs and K562-MVs (10 μg/mL) (top) and bar graphs for apoptosis rates (bottom); (**C**) The effects of MM-MVs on the morphology of the HK-2 cells treated with MM-MVs or K562-MVs (50 μg/mL) were observed using an charge-coupled Nikon Coolpix 995 digital charge-coupled device (CCD) camera attached to a Nikon Diaphot inverted phase-contrast microscope (Nikon, Tokyo, Japan), Magnification: 100×. * *p* < 0.05, ** *p* < 0.01, *** *p* < 0.001.

Severe acute kidney injury is very common in RI of MM (MM-RI) [3], and renal repair is dependent on tubular regeneration. To test whether MM-MVs might be involved into the development of MM-RI, we used MM-MVs to treat HK-2, a human proximal tubular cell line, and observed the changes in viability, apoptosis, and morphology in HK-2 cells. For analysis of cell viability, HK-2 cells (10^5^/mL) were cultured and treated with various concentrations of MM-MVs (0, 1, 5, 10, and 50 μg/mL) for 24, 48, and 72 h, respectively. We found that optical density (OD) values in HK-2 cells significantly decreased, after treatment of various concentrations of RPMI8226-MVs within 48 h in a dose-dependent manner (vs. control, *p* < 0.05). After 72 h, the decrease was significant in HK-2 cells treated with 50 μg/mL of RPMI8226-MVs, but not in the other groups (Figure 2A). In HK-2 cells treated with U266-MVs, the changes in OD value detected were similar to those in RPMI8226-MV-treated cells. However, MVs derived from K562 cells (K562-MVs), a human leukemia cell line, failed to exhibit this inhibitory effect (Figure 2A). Additionally, to exclude the possible effect of the mixed cytokines in the MV samples, we compared the effect of MM RPMI 8226 and U266 cell medium with and without the depletion of MV on the viability and apoptosis of HK-2 cells, respectively. No significant difference could be found between the two groups, suggested the mild effect of cytokines in the medium on the viability and apoptosis of HK-2 cells (Supplementary Figure S1).These results suggest that various concentrations of MM-MVs can significantly inhibit the viability in HK-2 cells, while this inhibitory effect reach to the utmost limit at 72 h even with the treatment of MM-MVs at 50 μg/mL (Figure 2A).

We next investigated the changes in apoptosis in HK-2 cells treated with various concentrations of MM-MVs at 48 h. It was shown that both types of MM-MVs significantly induced apoptosis in HK-2 cells in a dose-dependent manner (Figure 2B). Meanwhile, no obvious changes were observed in the HK-2 cells treated with various concentrations of K562-MVs (Figure 2B). These findings support that MM-MVs can induce apoptosis in HK-2 cells.

It has been reported that EMT plays an important role in tubulointerstitial renal fibrosis in MM [15]. Therefore, we observed the effect of MM-MVs on the morphology of the HK-2 cells treated with MM-MVs or K562-MVs (50 μg/mL) using an charge-coupled Nikon Coolpix 995 digital charge-coupled device (CCD) camera attached to a Nikon Diaphot inverted phase-contrast microscope (Nikon, Tokyo, Japan). As shown in Figure 2C, no distinct elongation/filopodia formation was found. This finding primarily suggests that MM-MVs did not induce EMT in the HK-2 cells.

### 2.3. MM-MVs Activate Apoptic Pathways of Caspase-3, -8, -9 and Bcl-2 Family Members

Based on the evident apoptosis in the HK-2 cells treated with MM-MVs, we next checked whether caspases, the key regulators of cell apoptosis, were involved in the induced effect. Additionally, as the essential regulators in caspase-mediated intrinsic apoptisis pathway, Bcl-2 family members, such as Bim, Bcl-xl, Bid, tBid, and Bcl-2, were investigated by Western blot.

**Figure 3:**
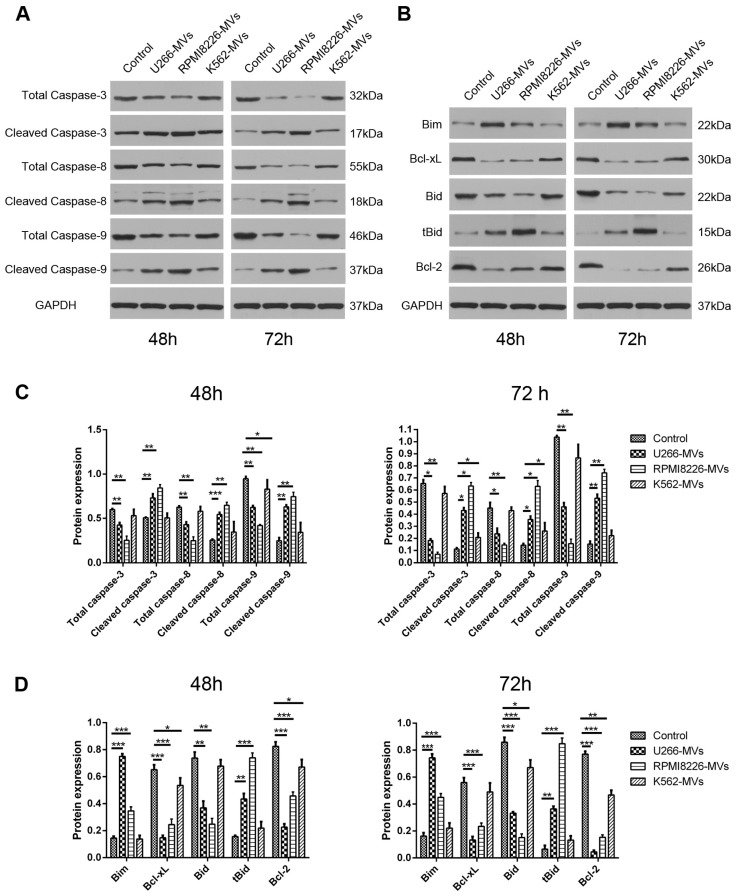
Activated apoptotic pathways of caspase-3, -8, and -9 and Bcl-2 family members in HK-2 cells treated with MM-MVs. HK-2 cells were treated with MM-MVs and K562-MVs (10 μg/mL) and the expression of proteins was assayed by Western blotting. (**A**,**C**) MM-MVs, but not K562-MVs, significantly induced the activation of caspase-3, -8, and -9. Both U266-MVs and RPMI8226-MVs reduced the expression of total caspase-3, -8, and -9, and increased the expression of cleaved caspase-3, -8, and -9; (**B**,**D**) MM-MVs up-regulated pro-apoptotic Bim and tBid proteins and down-regulated anti-apoptotic Bcl-xL and Bcl-2 proteins. Bar graphs for (**A**,**B**) were shown in (**C**,**D**), respectively. Each value was expressed as mean ± SD of three independent experiments. * *p* < 0.05, ** *p* < 0.01, *** *p* < 0.001 vs. control. 2.4. MM-MVs up-regulate E-cadherin protein and down-regulate vimentin protein in HK-2 cells.

HK-2 cells were incubated with different types of MVs at the concentration of 10 μg/mL for 48 or 72 h and the protein levels of caspase-3/8/9 and Bcl-2 family members were assayed. As shown in Figure 3A,C, both at 48 and 72 h, the cleaved caspase-3/8/9 levels were significantly up-regulated while their total caspase levels were down-regulated in both MM-MV groups, compared to the control and K562-MV group. Meanwhile, MM-MV treatment up-regulated the pro-apoptotic proteins (Bim and Bid) and down-regulated the anti-apoptotic proteins (Bcl-xl and Bcl-2) (Figure 3B,D). These results further evidence the MM-MV-induced apoptosis in HK-2 cells, which is possibly mediated by the mitochondrial-initiated intrinsic apoptotic pathways.

### 2.4. MM-MVs Up-Regulate E-Cadherin Protein and Down-Regulate Vimentin Protein in HK-2 Cells

**Figure 4:**
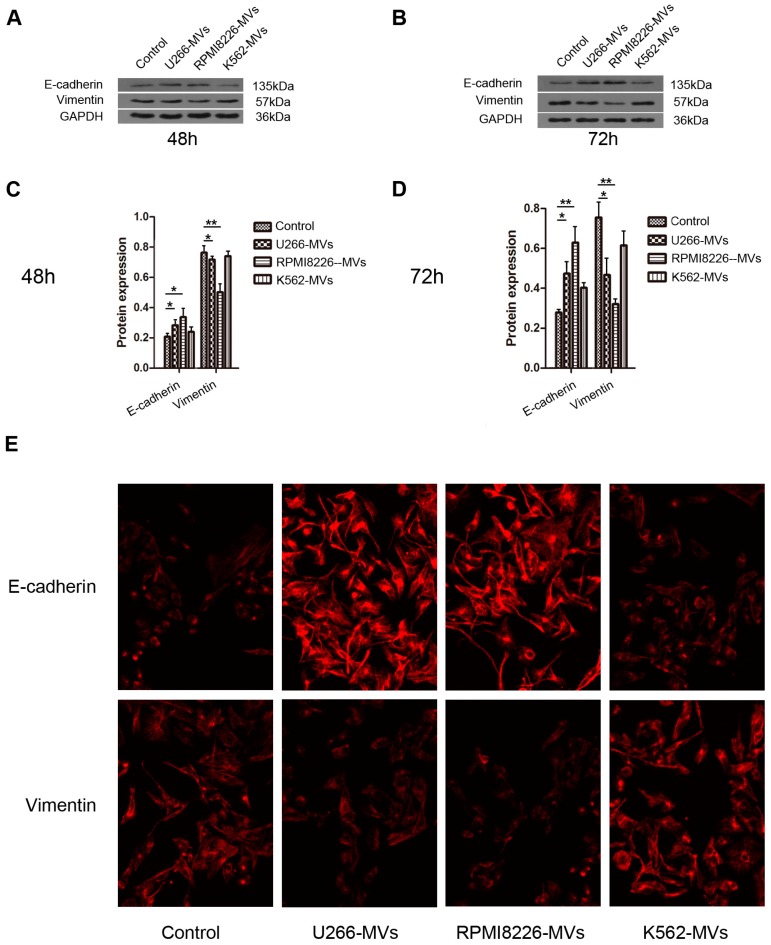
MM-MVs up-regulate E-cadherin protein and down-regulate vimentin protein in HK-2 cells. HK-2 cells were treated with MM-MVs and K562-MVs (10 μg/mL) and the expression of proteins was assayed using Western blot and immnofluorescence analysis. MM-MVs, but not K562-MVs, significantly up-regulated E-caderin protein and down-regulated vimentin protein in the HK-2 cells at 48 (**A**) and 72 h (**B**). Bar graphs for A and B were shown in (**C**) and (**D**), respectively. The two EMT markers were also assayed using a Nikon (Melvile, NY, USA) Eclipse TE300 fluorescence microscope in the HK-2 cells treated with MM-MVs and K562-MVs (10 μg/mL) for 72 h (**E**) magnification, 200×. Each value was expressed as the mean ± SD of three independent experiments. * *p* < 0.05, ** *p* < 0.01 vs. control.

To further study whether EMT was initiated in the MM-MV-treated HK-2 cells, HK-2 cells were incubated with different types of MVs at the concentration of 10 μg/mL for 48 or 72 h, and the changes in EMT markers of E-cadherin and vimentin were assayed using Western blot and immunofluorescence. As shown in Figure 4A–D, both at 48 and 72 h, MM-MVs significantly up-regulated E-cadherin levels and down-regulated vimentin levels in HK-2 cells, compared to the control and K562-MV group. However, K562-MVs showed no obvious effect on these EMT markers. Meanwhile, these findings were confirmed by immunofluorescence with staining of the primary antibodies (anti-E-cadherin and anti-vimentin, Figure 4E). Taken together, these findings confirm that MM-MVs can induce apoptosis, but not EMT in HK-2 cells.

### 2.5. Selective miRNAs in MM-MVs that Confer Caspase-3-Induced Apoptosis in HK-2 Cells

**Figure 5:**
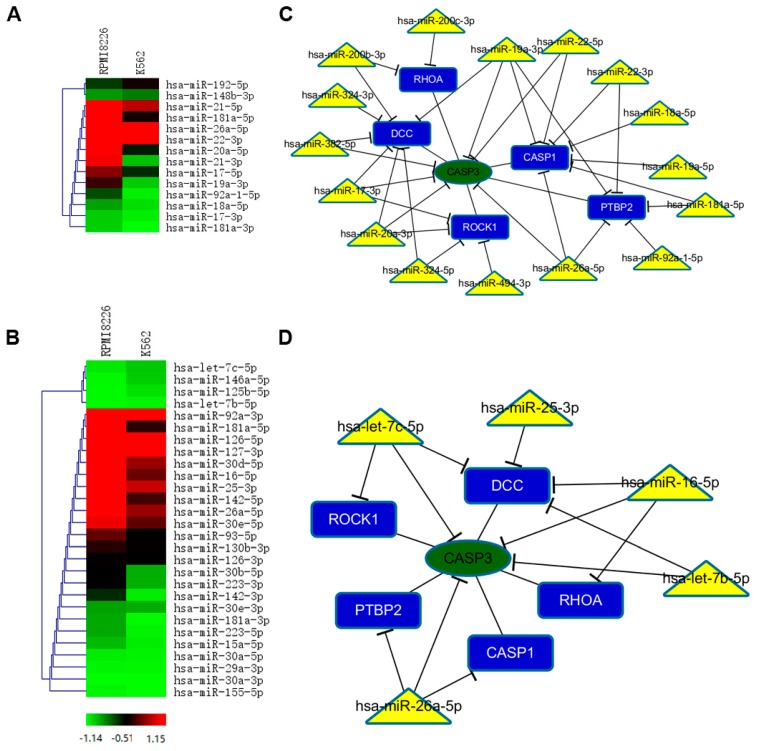

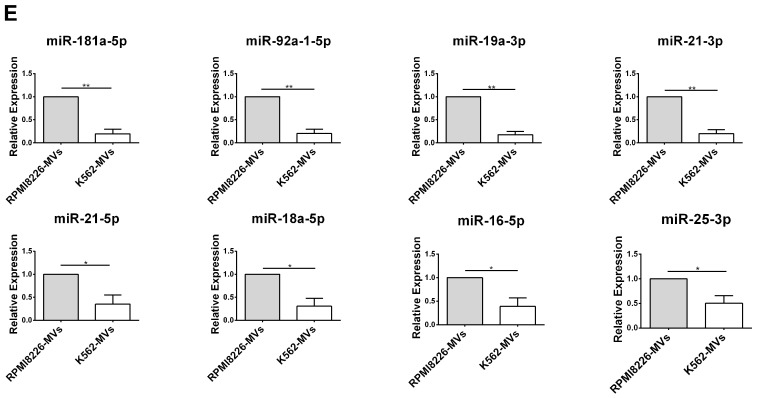
Selective miRNAs in MM-MVs that confer caspase-3-induced apoptosis in HK-2 cells. (**A**) Comparative analysis of renal-pathogenic miRNAs between RPMI8226-MVs and K562-MVs. RPMI8226-MVs were enriched in more highly-expressed renal-pathogenic miRNAs than did K562-MVs; (**B**) The heatmap of renal-protective miRNAs in RPMI8226-MVs and K562-MVs showed low levels of renal-protective miRNAs in the two types of MVs; (**C**,**D**) A schematic illustration representing MM-MV-packed miRNAs, including renal-pathogenic (**C**) and –protective miRNAs (**D**), and potential targets involved in regulation of caspase-3 in HK-2 cells. Yellow triangles: miRNAs relatively highly represented in RPMI8226-MVs. Green ellipse: caspase 3 gene. Blue rectangles: regulators interacted with hub genes and targeted by miRNAs; (**E**) Evaluation of the levels of the miRNAs involved in the regulation network and differentially expressed between RPMI8226-MVs and K562-MVs, using real-time PCR (* *p* < 0.01, ** *p* < 0.05).

As miRNAs are selectively excreted into MVs and act as the core elements for tumor cells [16], we next investigated the signature of miRNA repertoires in MM-MVs. Firstly, we performed small RNA-Seq analysis and focused on the expression of RI-related miRNAs. Intriguingly, the comparison of signature in RPMI8266-MVs and K562-MVs revealed the particular enrichment of these miRNAs. As shown in Figure 5A,B, several RI-related miRNAs (for example, miR-20a, miR-92a, miR-17, miR-19a, miR-18a, and miR-17) that had been ascertained in the published literature [17,18,19], were significantly highly represented in RPMI8266-MVs than in K562-MVs. In contrast, the documented renal-protective miRNAs, such as let-7b, miR-16, miR-25, and miR-29 family [20,21,22], were at extremely lower levels in both RPMI8226-MVs and K562-MVs. These results raised the possibility that a significant proportion of renal pathogenic miRNAs were selectively sorted into MM-MVs, consistent with a low expression of protective miRNAs. The signature of miRNA abundance might provide the role of secreted miRNAs in the inhibited viability and induced apoptosis in the HK-2 cells caused by MM-MVs.

Based on the induced expression of cleaved caspase-3 protein in the MM-MV-treated HK-2 cells, we further analyzed miRNAs in MM-MVs targeting caspase-3. We used collected RI-associated pathogenic and protective miRNAs to construct a caspase-3 regulating network. We found five important genes (*RHOA*, *DCC*, *CASP1*, *PTBP2*, and *ROCK1*) interact with caspase-3 directly. In the pathogenic miRNAs caspase-3 network (Figure 5C), 16 miRNAs contribute to the caspase-3 regulatory network, six miRNAs (hsa-miR-382-5p, hsa-miR-17-3p, hsa-miR-20a-3p, hsa-miR-26a-5p, hsa-miR-22-5p, and hsa-miR-19a-3p) repress caspase-3 directly, and the rest of the miRNAs regulate five other genes to affect caspase-3 indirectly. Similarly in Figure 4D, four protective miRNAs interact with caspase-3 directly. There are more pathogenic miRNAs than protective miRNAs contributing to regulate caspase-3. Furthermore, we evaluated the levels of the miRNAs that were involved in the regulation of caspase-3 and differentially expressed between RPMI8226-MVs and K562-MVs, using single assay real-time PCR (Figure 5E).

Though the other activated apoptosis regulators were not similarly investigated, these findings demonstrate that RI-associated miRNAs are selectively enriched in MM-MVs and might contribute to the induced apoptosis in the HK-2 cells by MM-MVs.

### 2.6. CD138+ Circulating MV (cirMV) Counts Positively Correlate with Renal Impairment

**Figure 6:**
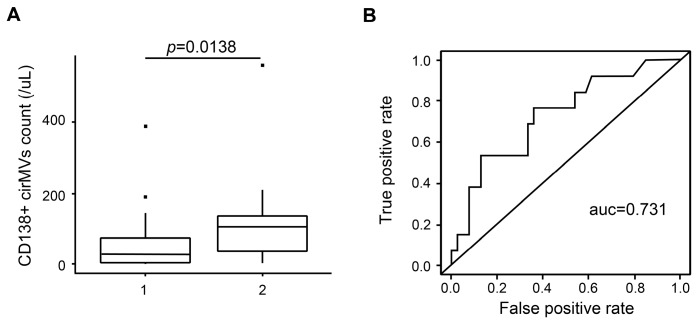
CD138+ cirMV counts positively correlate with renal impairment. The enrolled 61 de novo patients with MM were divided into two groups according the levels of SCr (group 1, SCr < 2 mg/dL, *n* = 45; group 2 SCr ≥ 2 mg/dL, *n* = 16). The levels of CD138+ cirMVs were significantly higher in group 1 than in group 2 (**A**, *p* = 0.0138), and the ROC value for using CD138+ cirMV counts to diagnose RI in de novo patients with MM was 0.731 (**B**).

Due to the effects of MM-MVs on HK-2 cells, we further investigated the clinical significance of MM-MVs in patients with MM. In this study, we enrolled 61 de novo patients with MM (Table 1), and circulating MVs (cirMVs) in the peripheral blood were analyzed using a BD LSR II flow cytometer (BD Biosciences, San Jose, CA, USA) equipped with FACSDiva software. Based on the phenotypic characteristics of MM-MVs and the findings by Krishnan and collegues [23], we used CD138+ cirMVs to represent circulating MM-MVs in patients with MM. Meanwhile, according to the serum creatinine levels (SCr), the patients were classified into two groups (group 1, SCr < 2 mg/dL, *n* = 45; group 2, SCr ≥ 2 mg/dL, *n* = 16). We assayed the association between RI and CD138+ cirMV counts in the two groups. We found that CD138+ cirMV counts were significantly higher in group 1 than in group 2 (*p* = 0.0138), and the ROC value for using CD138+ cirMV counts to diagnose RI in de novo patients with MM was 0.731 (Figure 6). These findings support that CD138+ cirMV counts play a role in the diagnosis of RI in de novo MM patients.

## 3. Discussion

Mayo Clinic [24] reported that both Ig-relevant and Ig-irrelevant damage exist in MM-associated RI, the underlying mechanisms remain elusive. Here, our data demonstrate that MM-MVs significantly inhibit viability and induce apoptosis, but not EMT in HK-2 cells, and our analysis of the signature of miRNA repertoires in MM-MVs support the miRNA content might potentially contribute to these effects. Furthermore, we confirmed the clinical significance of peripheral circulating CD138+ cirMV counts in RI in de novo MM patients. To the best of our knowledge, this is the first study to investigate the role of MM-MVs in myeloma RI.

MiRNAs are small noncoding RNAs (19–24 nucleotides in length) that down-regulate gene expression at the post-transcriptional level, and play crucial roles in diverse physiological processes and certain miRNAs have been demonstrated involving in kidney physiology and pathophysiology [25]. MV-mediated transfer of miRNAs, as a degrading enzyme-protective way, has been verified in various contexts. For example, recent reports have demonstrated that miRNAs carried by MSC-MVs can facilitate renal recovery from acute kidney injury [26,27]. Here, small RNA sequencing analysis of MVs exhibited that renal-pathogenic miRNAs were more abundant in RPMI8226-MVs than K562-MVs. In particular, miR-21 [28], miR-181 [29], and miR-20 [30] had been demonstrated as key regulators in pathological processing of renal disease. On the contrary, renal-protective miRNAs were expressed at low levels in RPMI8226-MVs. Thus, we considered the aberrant expression of renal-disease related miRNAs in MM-MVs might contribute to the adverse effects on HK-2 cells. The exact roles of the specific miRNA or all of the miRNAs are to be investigated.

Our in vitro study demonstrates that RPMI8226-MVs and U266-MVs inhibited viability in HK-2 cells, in large part due to the enhanced cell apoptosis. However, the viability in the untreated HK-2 cells showed mild changes within the three days of culture, which might be due to the high cell density, and the inhibitory effect of MM-MVs on HK-2 viability seemed to reach the utmost limit at 72 h, which implies that MM-MVs should be added more than once, or there are unknown mechanisms underlying the resistance. It is known that caspase-8/9 are important initiators for extrinsic and intrinsic apoptosis. In these two kinds of apoptosis, the cleaved caspases mediated the activation of caspase-3, which is a critical executive molecule in apoptosis and has been demonstrated and applied regularly in apoptosis detection [31]. Our results showed that the caspase-3/8/9 were activated either by increasing the MM-MV concentration or prolonging the incubation time, suggesting that MM-MVs accelerate apoptosis, possibly via caspase signaling in HK-2 cells. Actually, the results confirmed that Bcl-2 family members were involved, with the up-regulated expression of pro-apototic regulators of Bim and Bid, and the down-regulated expression of anti-apototic regulators of Bcl-xl and Bcl-2. As MV-shuttled miRNAs are complex populations we considered, instead of a single miRNA determinant, that the complicated interactions of the molecular landscape contribute to the function of MM-MVs, which had been shown in the miRNA regulation networks. To be noted, our clinical data of 61 de novo patients demonstrate that CD138+ cirMV counts were positively correlated with the severity of renal impairment, suggesting of the potential role of MM-MVs as a driver biomarker in the development and diagnosis of RI of MM patients. It is certainly critical to directly assay the effect of primary MM-MVs from MM patients on HK-2 viability and apoptosis, and to deeply study their miRNA content. However, because of the difficulties in isolating and purifying enough CD138+ cirMVs from the limited volume of peripheral blood, we failed to present these data here. In addition, the functions, signaling pathways, and critical determinants variations in recipient cells underlying our findings are also challenges promising to be solved in the future.

## 4. Materials and Methods

### 4.1. Cell Culture

Human RPMI8226 and U266 MM cell lines (purchased from American Type Culture Collection, ATCC, Rockefeller, MD, USA), human chronic myeloid leukemia cell line K562 (routinely preserved in our laboratory), and human renal tubular epithelial cell line, HK-2 cells (purchased from Guangzhou Jennio Biological Technology, Guangzhou, China) were cultured in RPMI-1640 containing 10% fetal bovine serum (FBS; Hyclone, South Logan, UT, USA), and 1% penicillin-streptomycin (Beyotime, Shanghai, China) at 37 °C in a 5% CO_2_ humidified atmosphere.

### 4.2. MV Isolation and Quantification

MVs were isolated by differential ultracentrifugation as we described previously [11,14]. Briefly, harvested cell culture medium was centrifuged at 750× *g* for 15 min and 1500× *g* for 20 min to remove cells debris. Then MVs were pelleted. The supernatant was discarded and the MV pellet was washed and resuspended in PBS, followed by another centrifugation at 16,000× *g* for 1 h at 4 °C. The quantity of MVs was determined by measuring the total protein content through the BCA protein assay kit (Beyotime, Shanghai, China). The MVs were prepared before use.

### 4.3. Transmission Electron Microscopy (TEM) and Scanning Electron Microscopy (SEM)

TEM (FEI, Hillsboro, OR, USA) was conducted as per our published papers [14]. MM-MVs were also observed by SEM (FEI, Hillsboro, OR, USA). Poly lysine coated coverslip preparation, cell fixation, dehydration, drying, and coating were performed as per the regular protocol [32]. Images were captured with a scanning electron microscope (Tescan, Brno, Czech Republic).

### 4.4. Small RNA Sequencing and Data Analyses

Total RNA of RPMI8226-MVs was used to generate a small RNA sequencing library using reagents and methods provided with TruSeq Small RNA Sample Prep Kit (Illumina, San Diego, CA, USA) and sequenced by IlluminaHiSeq. The K562-MVs small RNA-seq data were from our previous findings [33]. The FASTQ files of the RNA-Seq and small RNA-Seq data of RPMI8226-MVs and K562-MVs were submitted to the NCBI Sequence Read Archive (SRA) under accession numbers SRP092289 and SRP057826, respectively. We trimmed the 5′ and 3′ adapters, filtered the low-quality reads, and kept the 18~30 nt reads. The reads were mapped to hg19 with BWA [34]. The remaining reads were mapped to human pre-miRNA miRBase release 20 [35] with miRExpress 2.0 [36]. The expression level of miRNA was normalized with RPM (reads per million mapped reads).

### 4.5. Cell Viability Detection

The viability analysis of HK-2 Cells treated by MM-MVs was performed by Cell Counting Kit-8 (CCK-8, Guangzhou Yiyuan Biotech. Co., Ltd., Gunagzhou, China). HK-2 cells were incubated with various MVs (RPMI 8226, U266, and K562-MVs) at a range of concentrations (0, 1, 5, 10, and 50 μg/mL) for different times (24, 48, or 72 h). Incubation accomplished, the fresh CCK working liquid (dilution 1:10) was added and incubated for 2.5 h. The absorbance value was determined at 450 nm using a microplate reader.

### 4.6. Apoptosis Detection

Cell apoptosis rate was measured by flow cytometry (FCM) using the Annexin V–PI Apoptosis Detection Kit (Beyotime, Shanghai, China). HK-2 cells were placed at 1 × 10^5^/mL in triplicate. In 24 h, culture media was discarded and cells were resuspended with MV suspension at different concentration. For the medium groups, HK-2 cells were incubated in six-well plates with 100 μL/well cultured medium of MM cells, with or without the depletion of MVs. The treated HK-2 cells were cultured for 48 or 72 h, then an Annexin V-PI apoptosis detection kit was used according to the manufacturer’s instructions. Afterwards, the apoptosis analysis was conducted using a FACSAria II (BD Pharmingen, San Diego, CA, USA).

### 4.7. Western Blotting

HK-2 cells were treated with 10 μg/mL MVs of various cell types and collected at 48 and 72 h. Then, cells were solubilized in sodium dodecyl sulfate lysis buffer. Protein samples were separated by sodium dodecyl sulfate polyacrylamide gel electrophoresis (SDS-PAGE), transferred onto a phenylmethylsulfonyl fluoride (PVDF) membrane, and incubated with the primary rabbit anti-human total caspase-3, cleaved caspase-3, total caspase-8, cleaved caspase-3, total caspase-9, cleaved caspase-9, Bim, Bcl-xl, Bid, cleaved Bid, Bcl-2, E-cadherin, Vimentin, and GAPDH antibodies (Sigma-Aldrich, St Louis, MO, USA) at 4 °C. After washing with TBS-T five or six times, the membranes were then probed with horseradish peroxidase (HRP)-conjugated secondary goat anti-rabbit antibody (Wuhan Boster Biotechnology Co., Ltd., Wuhan, China) for 2 h at room temperature. Enhanced chemiluminescence was performed according to the manufacturer’s instructions (Beyotime, Shanghai, China). All the experiments were repeated in triplicate.

### 4.8. Immunofluorescent Stainning

After treatment of MVs (10 μg) for 72 h, HK-2 cells were cultured on sterile glass coverslips and fixed in 4% paraformaldehyde for 30 min. After washing with PBS three times, the coverslips were permeabilized with 0.1% Triton X-100 for 5 min, blocked in 5% bovine serum albumin (BSA), and then incubated with the primary antibodies (anti-E-cadherin and anti-vimentin, TDY, Beijing, China) at 4 °C overnight. The slips were next washed and incubated with Cy-3-conjugated goat anti-mouse or anti-rabbit secondary IgG (Proteintech Group, Rosemont, IL, USA) at room temperature for 1 h. Cells were observed using a Nikon (Melvile, NY, USA) Eclipse TE300 fluorescence microscope.

### 4.9. Construction of miRNA Regulatory Network

Known miRNAs related to renal disease are important for uncovering the co-regulation of the renal disease. We collected valid miRNAs in renal disease through reviewing peer-reviewed publications. We screened differentially-expressed miRNA from known miRNAs datasets labeled as “pathogenic” and “protective”, and obtained their predicted targets by the Targetscan [37] and miRanda [38]. Genes and candidate miRNAs were constructed regulating network by Cytoscape [39].

### 4.10. Realtime PCR

We used Trizol reagent (Invitrogen, Waltham, MA, USA) to isolate total RNA from RPMI8226-MVs and K562-MVs according to the manufacturer’s instructions. RNA quality and purity were determined by a nucleic acid/protein analyzer (Beckman Coulter, Brea, CA, USA), with the A_260_/A_280_ ratio values ranging from 1.8 to 2.0. Reverse transcription was performed by the FastQuant RT Kit (TIANGEN, Beijing, China), and then the real-time reverse transcriptase polymerase chain reaction (RT-PCR) was conducted with the SYBR green PCR master mix (TIANGEN) on an Applied Biosystems 7500 Real-Time PCR System. Each reaction was performed in triplicate and normalized to U6. Relative expression levels of miRNA were expressed through the ΔΔ*C*_t_ method.

### 4.11. Clinical Samples Collection

The peripheral blood (PB) of MM patients was obtained in anti-coagulate tubes with ethylenediaminetetraacetic acid (EDTA) anticoagulant, which has been considered more appropriate than heparin for MV detection by flow cytometry in our previous study [40]. All MM patients were from Institute of Hematology, Union Hospital, Tongji Medical College, Huazhong University of Science and Technology. This study was approved by the Review Board of Tongji Medical College (No. 2012S119, 6 March 2012) and informed consent was obtained from each volunteer. The EDTA-anticoagulated blood was double centrifuged at 2500× *g* for 30 min at 20 °C. Platelet-free plasma was obtained and then centrifuged at 16,000× *g* for 15 min at 20 °C to harvest cirMVs.

### 4.12. Quantitative Detection of cirMVs

The detailed protocol of MVs enumeration by FCM has been stated in our previous reports [40]. We defined MM-cirMVs as CD138+Calcein-AM+vesicles with a diameter of less than 1 µm. The cirMVs preparation was resuspended by PBS, and then stained with calcein-AM and CD138-PE. After incubation for 30 min at 4 °C, cirMVs were washed with PBS at 16,000× *g* for 15 min and resuspended for examination by a FACSAria II (BD Pharmingen, San Diego, CA, USA).

### 4.13. Statistical Analysis

GraphPad Prism version 6.0 (GraphPad Software, GraphPad Software Inc, California, KY, USA) and R version 3.2.2 were used for all calculations and figures. Nonparametric and unpaired *t*-tests were used to compare groups. The Wilcoxon rank test was used for comparisons of cirEV counts between the two groups of patients with MM. ROC curves were used to determine the sensitivity, specificity, and positive and negative predictive values. All presented *p*-values are two-sided, and *p* < 0.05 was considered to indicate statistical significance.

## 5. Conclusions

In conclusion, this primary study supported MM-MVs to represent a previously undescribed factor and play a potential role in the development of the characteristic renal lesion in kidney damage of MM patients. Our study sheds light on the potential application of CD138+cirMV counts in precise diagnosis of RI in MM and exploring MM-MVs as a therapeutic target.

## Figures and Tables

**Table 1:** Clinical characteristics of patients with de novo multiple myeloma (MM).

Clinical Characteristics	Patient Number (*n*)
Total	61
Sex	
Male	40
Female	21
Median age, years (range)	59 (40–82)
Durie-Salmon stage	
I	3
II	11
III	38
International staging system	
I	6
II	25
III	27
Type of monoclonal Ig	
IgG	27
IgA	16
IgD	3
LC ^a^ only	8
Renal function	
Group 1 (SCr ^b^ < 2 mg/dL)	45
Group 2 (SCr ≥ 2 mg/dL)	16

^a^ LC, light chain; ^b^ SCr: serum creatinine.

## Supplementary Materials

Supplementary materials can be found at www.mdpi.com/1422-0067/18/3/513/s1.

## Abbreviations

RI	Renal Impairment
MM	Multiple Myeloma
MVs	Microvesicles
EMT	Epithelial-Mesenchymal Transition
RPM	Reads Per Million Mapped Reads
