# Supplementary material for: Tumor Cell-Derived Microvesicles Induced Not Epithelial-Mesenchymal Transition but Apoptosis in Human Proximal Tubular (HK-2) Cells: Implications for Renal Impairment in Multiple Myeloma
Source: Int J Mol Sci. 2017 Feb 27;18(3):513. doi: 10.3390/ijms18030513 (PMC5372529; doi:10.3390/ijms18030513)
Supplement: Supplementary file 1 [file ijms-18-00513-s001.pdf]

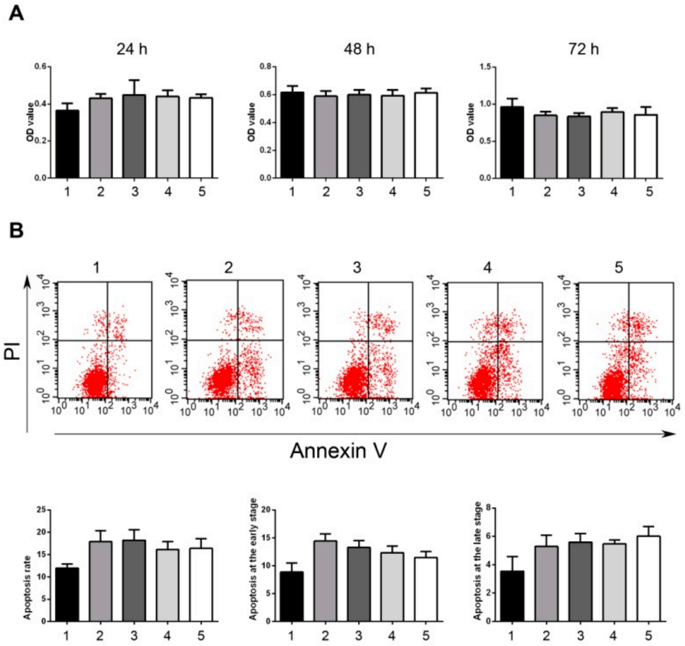

Figure S1. The effect of MM cell medium with or without the depletion of MVs on cell viability and apoptosis in HK-2 cells. (**A**) HK-2 cell viability was determined with CCK-8 assay. No statistical differences were found among the groups; (**B**) Representative FCM analysis of HK-2 cell apoptosis after 48 h of treatment (top) and bar graphs for apoptosis rate (bottom). No statistical differences were found among the groups. 1: control; 2: U266 medium; 3: U266 medium without MVs; 4: RPMI8226 medium; and 5: RPMI8226 medium without MVs.
